# Comparison of SNP and CAPS markers application in genetic research in wheat and barley

**DOI:** 10.1186/s12870-015-0689-9

**Published:** 2016-01-27

**Authors:** Yuri Shavrukov

**Affiliations:** School of Agriculture, Food and Wine, University of Adelaide, Adelaide, Australia; Department of Biological Sciences, Flinders University, Adelaide, Australia

**Keywords:** Barley, CAPS, high-throughput technology, molecular markers, SNP, SNP frequency, wheat

## Abstract

**Background:**

Barley and bread wheat show large differences in frequencies of Single Nucleotide Polymorphism (SNP) as determined from genome-wide studies. These frequencies have been estimated as 2.4-3 times higher in the entire barley genome than within each diploid genomes of wheat (A, B or D). However, barley SNPs within individual genes occur significantly more frequently than quoted. Differences between wheat and barley are based on the origin and evolutionary history of the species. Bread wheat contains rarer SNPs due to the double genetic ‘bottle-neck’ created by natural hybridisation and spontaneous polyploidisation. Furthermore, wheat has the lowest level of useful SNP-derived markers while barley is estimated to have the highest level of polymorphism.

**Results:**

Different strategies are required for the development of suitable molecular markers in these cereal species. For example, SNP markers based on high-throughput technology (Infinium or KASP) are very effective and useful in both barley and bread wheat. In contrast, Cleaved Amplified Polymorphic Sequences (CAPS) are more widely and successfully employed in small-scale experiments with highly polymorphic genetic regions containing multiple SNPs in barley, but not in wheat. However, preliminary ‘*in silico*’ search databases for assessing the potential value of SNPs have yet to be developed.

**Conclusions:**

This mini-review summarises results supporting the development of different strategies for the application of effective SNP and CAPS markers in wheat and barley.

## Background

Single Nucleotide Polymorphism (SNP), the substitution of a single nucleotide in any part of the genome as a result of natural mutation, has become one of the most powerful tools in molecular biology (Reviewed in [1]). Large-scale genome reorganisations such as translocations, duplications or substantial deletions/insertions are very often eliminated by natural selection, except in rare cases where the change provides a direct benefit for the mutated organisms. One example of this is plant genome polyploidisation in adverse environments (Reviewed in [2]). In contrast, SNPs as point mutations can be evolutionally neutral and escape the pressure of natural selection if it occurs in non-coding regions or does not affect amino acid sequence of the encoded polypeptides. Subsequently, SNPs have become widely distributed in genomes of all living organisms (Reviewed in [3]).

In plants, SNPs have a particularly important application as molecular markers reflecting both natural genetic variability and a genetic drift created by breeders during the course of crops improvement (Reviewed in [4–9]). The application of SNP markers has shown rapid progress in recent years that technological advances and the expansion of low cost services have made sequencing a routine practice widely available to scientists. The presence of SNPs among parents of segregating populations or in a panel of genotypes is important factor when choosing the most suitable strategy for genetic polymorphism analysis. Many different types of molecular markers are based on SNP identification and each has accompanying advantages and disadvantages. In the present mini-review we compare high-throughput technology of SNP markers and Cleaved Amplified Polymorphic Sequences (CAPS) markers in regard to their application in the two important cereal crops of wheat and barley.

### High-throughput SNP analysis: Illumina vs. KASP technology

The work of many researchers focuses on individual genes that are of significance to their area of study. Such Gene of Interest (GOI) can be sequenced and genetic polymorphism within the sequences can then be identified and analysed manually. However, large-scale Whole Genome Sequencing (WGS) is now achievable through robotics high-throughput technology. Thousands of SNP markers can be identified at a time and analyse hundreds of accessions. Initial applications of SNP markers were based on the high-throughput platform from the American company Illumina that was very suitable for plants [5]. Our own experience of Next Generation Sequencing (NGS) with 9 K Illumina Infinium SNP array in wheat [10] confirmed the supreme effectiveness of this technology in crops [11]. In wheat, the numbers of available SNP markers is growing rapidly jumping from 90 K as recorded by the new Infinium [12, 13] to 500 K and 4 M in Illumina shortgun WGS array [14, 15].

Many of the SNP markers identified by Illumina Infinium or shortgut WGS arrays have already been mapped out in the chromosomes. In conjunction with the easy to use and publicly available software FlapJack (http://www.hutton.ac.uk/research/groups/information-and-computational-sciences/tools), SNP can be clearly visualised in the linear arrangement that allows a direct comparison of genotypes, as presented earlier [10].

Despite the wide distribution of NGS Infinium and WGS shortgun from Illumina, the British company LGC Genomics has developed an alternative high-throughput technology named KASPar or KASP [16]. “The KASP assay utilizes a novel homogeneous fluorescent genotyping system” [16], that claims to provide greater flexibility for researchers. KASP technology has been successfully utilised for the SNP analysis of pigeonpea [17], peanut [18] and soybean [19]. The comparison of KASP markers to other methods of SNP genotyping has been reviewed in: [6–9, 11].

High-throughput methods cannot be easily adapted to the study of GOI, where SNPs must be found and assessed on a very short fragment of the coding region. Most often, researchers will instead sequence the full gene and later amplify a certain genetic fragment manually. The only option for the use of high-throughput automatic systems is to study multiple accessions or progeny segregations using a very limited number of primer-sets. In this case, a service providing a molecular analysis of DNA samples using various high-throughput SNP technologies can be applied.

### CAPS markers as an example of manual SNP analysis

Many small-scale laboratories are focused on a single or very limited numbers of GOI. During sequencing, there can be no guarantee that an identified SNP will be suitable for use as a molecular marker. In plant biology, a common practice is to design one primer so that the 3’-end is exactly located on the SNP position. Depending on the match or mismatch of the SNP in the 3’-end of the primer, a positive or null-band from PCR amplification will then be produced. This approach is named Allele-Specific PCR (AS-PCR) and has been successfully used in plant biology [11, 20].

However, if an SNP occurs within the recognition site of a restriction enzyme, it is much easier to use CAPS markers. The digestion of PCR products and subsequent separation of the fragments in agarose gel is a simple approach that can be carried out in any laboratory with basic molecular equipment, achieving accurate and clear results. The recent book [21] and review [22] compiled and discussed hundreds of examples of CAPS markers and their application in plant biology.

### SNP and CAPS in wheat and barley

The high-throughput technology of SNP markers is very effective in bread wheat (*Triticum aestivum* L.). As mentioned above, 9K and 90K SNP arrays are now routinely employed for bread wheat analyses [10, 11, 13, 23]. In durum wheat, the range of applied SNP markers is even wider including: 2.6K [24], 26K [25] and 90K [12]. Most recently, a 9M SNP array in a single homeologous group of chromosome 7 in bread wheat has been reported [15]. Clearly, SNP markers continue to be of great value to plant genetic research and the true extent of their worth may not yet be apparent.

In cultivated barley (*Hordeum vulgare* L.), the reported number of SNP markers is quite variable: 1.5K [26], 4.5K [27], 9K [28], and 22K [29]. The barley genome is estimated to be about 5.1 Gb with 26,159 ‘high-confidence’ genes recognised [30]. This is smaller than each of three genomes of hexaploid wheat at an estimated total of 17 Gb, with 124,201 identified gene loci [31]. Nevertheless, high-throughput technology using SNP markers is also very effective in barley [26–29], so we can conclude that there is no appreciable difference with SNP marker application in wheat and barley.

However, the same cannot be applied to CAPS markers. Many reports have indicated that the application of CAPS markers is much simpler and more productive in barley than in wheat (Reviewed in [20, 22]). A number of groups have shown success in developing CAPS markers for Marker-assisted selection in barley [32–34]. In bread wheat, CAPS markers are reportedly much rarer [35–37] but they have been found in tetraploid *Triticum dicoccoides* [38] and *Aegilops tauschii* Coss, the wild progenitor of D genome [39].

The difference in the expected frequencies in both crops can be illustrated by the rather unusual example of restriction enzyme selection. In this case, scientists working with a specific GOI in barley did not sequence the amplified PCR fragments at all. In the absence of a genetic sequence, Řepková et al. [40] digested a 511-bp PCR product amplified from the powdery mildew resistance gene, *Mla*, with 12 different restriction enzymes. Presumably, the choice of restriction enzymes was based on their availability in the laboratory and was otherwise random. Two restriction enzymes (*Dra*I and *Hpa*II), with completely non-related recognition sites, revealed a polymorphism between resistant and sensitive barley plants. *Hpa*II was chosen for CAPS marker development, eventually leading to its successful application [40]. This demonstrates how effective CAPS markers can be easily identified using even an economically non-optimal method in barley. However, such a strategy is most unlikely to be successful in wheat, where the occurrence of SNPs is much rarer, and thus is more similar to the probability of winning ‘the Jackpot’ in a lottery. In wheat, a known sequence with one or more identified SNPs is essential for CAPS markers development.

The differences in SNP and CAPS markers in barley and wheat are summarised in Table 1a. SNPs are used as high-throughput derived markers, effective in both wheat and barley. However, CAPS markers, most suitable for manual application, showed excellent results in barley but very poor results in wheat (Reviewed in [20]). A topic of great interest when examining the differences observed in the frequencies of SNPs in both barley and wheat is to debate the possible biological origins behind these differences, as is discussed in the following section.

**Table 1:** Comparison of SNP and CAPS markers application in wheat and barley (a), and corresponding frequencies of ‘true’ SNPs in genomes (b)

	Barley	Bread wheat
A. Effectivity of markers		
SNP	Very good, high-throughput	Very good, high-throughput
CAPS	Very good, manual operation	Poor, manual operation
B. Frequencies of SNPs (one ‘true’ SNP per number of bp^a^)		
Entire genome	189-240	540-569
GOI^b^	7-64	335-813

^a^Base pair, ^b^Gene of Interest.

### Comparison of SNP frequencies in barley and wheat

In barley, the SNP frequencies determined from genome-wide studies or at least multiple gene surveys record one SNP per 240 bp [41, 42], per 200 bp [43] and per 189 bp [44]. In contrast, barley SNPs within individual barley GOI may be significantly more frequent than quoted. For example, roughly one SNP is present per 64 bp in the β-amylase *Bmy1* gene [45], per 42 bp in the scald resistance *Rrs2* gene [46], per 29 bp in aluminium tolerance gene, *HvMATE1* [47], per 27 bp in the intronless *Isa* gene [48], and per 7 bp in the leaf rust resistance *Rph7* gene [49] (Table 1b).

Hexaploid wheat is an allopolyploid species with three different genomes (A, B and D). SNPs identified among homoeologous sequences in these three genomes are named ‘false SNP’ [24] and reflect interspecific genetic differences among ancestors of the three genomes in wheat. The frequencies for such ‘false SNP’ in bread whet are quite high and are reported as one SNP per 20 bp [50], per 24 bp [51] and up to 61 bp [52]. In contrast, intervarietal polymorphisms among homologous chromosomes of different genotypes are named ‘true SNPs’ [24]. An entire wheat genome analysis of bread wheat reported one ‘true SNP’ per 540-569 bp [11, 12, 51] within one of three genomes. Interestingly, the SNP frequency in GOI was relatively similar to the number recorded in the entire genome: one ‘true SNP’ per 335 bp in 21 studied genes [53], one SNP per 556 bp in the Grain Protein Content B1 gene, *GPC-B1* [54], and one SNP per 613 bp in 13 studied genes [55] (Table 1b).

SNP marker analysis in barley and bread wheat (Table 1a) to detect ‘true’ SNP frequencies in entire genomes (Table 1b), has ascertained some 2.4-3-fold more SNPs in barley compared to wheat. Despite a high overall efficiency in high-throughput SNP analyses, when considering specific GOI the frequencies of SNPs are very different (Table 1b), namely 5.2-87.6-fold higher in the genome of barley than wheat. Furthermore, it was reported that wheat has the lowest level of useful SNP-derived markers while barley has been estimated to contain the highest level of polymorphism [52]. Because the detection of SNPs in GOI is the most critical step for CAPS markers developments, we can conclude that the enormous differences in SNP frequencies in GOI between barley and bread wheat is the main reason for variability in the results of CAPS in these crops (Table 1a). The biological basis for the phenomenon is likely based upon the evolutionary origin of both crops.

### Evolutionary differences in wheat and barley

Genetic differences between wheat and barley arise from the individual origins and evolutionary history of both species. Bread wheat contains rare SNPs as a result of the double genetic ‘bottle-neck’ created by the natural hybridisation and spontaneous polyploidisation that led to a significant reduction of genetic polymorphisms. Recent data has revealed that the initial hybridisation between progenitors of A and B genomes occurred between 0.52-0.82 million years ago [56], significantly earlier than was initially proposed [57, 58]. Since that time, A and B genomes have co-evolved in the genomes of tetraploid wheat. The second event of hybridisation with *Aegilops tauschii* (D genome) is estimated to have taken place about 8-9 thousand years ago [59–61].

The size of each separate genome, A, B or D, in bread wheat is comparable to the size of the genome in diploid barley. However, the percentage of non-coding genetic regions on the chromosomes with repetitive elements is dramatically different in bread wheat and barley, accounting for more than 85 % of the wheat genome [11]. The domestication of wheat and barley occurred in parallel in ancient times, but lower frequencies of SNPs were more common in bread wheat, while domesticated barley remains more polymorphic species [59, 61].

Cultivated barley also experienced a genetic ‘bottle-neck’ through domestication, but the breeding pressure was less strong than in wheat resulting in more frequent polymorphisms [27]. The majority of the genetic variation in genepool of modern elite barley genotypes can be assessed with 100-1000’s of robust markers such as SNPs [27]. Therefore, significantly less applied SNP markers in barley revealed similar genetic polymorphism compared to wheat.

## Conclusions

In summary, different strategies are required for the development of the most suitable molecular markers in the cereal species. High-throughput technology is very effective for SNP marker development in both bread wheat and barley despite considerable differences in the rate of their occurrence in the entire genomes: 2.4-3-fold more in barley than in each of three genomes of wheat. The potential SNPs for either Infinium or KASP high-throughput technology have to be initially searched ‘*in silico*’ in different databases following assessment of effective SNPs. Clear differences between barley and bread wheat are shown in the application of manually developed CAPS markers. In barley, the presence of highly polymorphic genetic regions containing multiple SNPs allows the simple development of CAPS in small-scale experiments. However, it is a much harder task to develop CAPS in bread wheat due to significantly lower occurrence of SNPs (5.2-87.6-fold in GOI).
